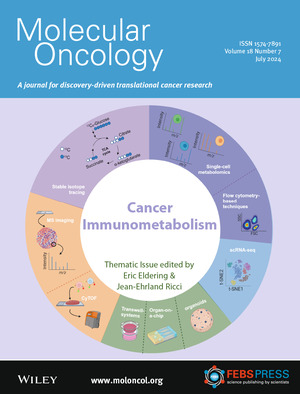# Issue Information

**DOI:** 10.1002/1878-0261.13461

**Published:** 2024-07-04

## Abstract

Metabolic rewiring within the tumour microenvironment modulates not only the function of host immune cells, but also the capacity of tumour cells to grow and respond to immunotherapy. The ‘Cancer Immunometabolism’ thematic issue explores the therapeutic modalities related to immunometabolism in cancer treatment.

**On the cover:** State‐of‐the‐art techniques to unravel immune and metabolic heterogeneity as well as spatial resolution in the context of inflammatory microenvironments. Read the Review article by Verheijen et al, in pp. 1759–1776.

**Illustration credits:** Image created with BioRender.com